# Multivesicular Release Underlies Short Term Synaptic Potentiation Independent of Release Probability Change in the Supraoptic Nucleus

**DOI:** 10.1371/journal.pone.0077402

**Published:** 2013-09-24

**Authors:** Michelle E. Quinlan, Michiru Hirasawa

**Affiliations:** Division of Biomedical Sciences, Faculty of Medicine, Memorial University, St. John’s, Newfoundland, Canada

## Abstract

Magnocellular neurons of the supraoptic nucleus receive glutamatergic excitatory inputs that regulate the firing activity and hormone release from these neurons. A strong, brief activation of these excitatory inputs induces a lingering barrage of tetrodotoxin-resistant miniature EPSCs (mEPSCs) that lasts for tens of minutes. This is known to accompany an immediate increase in large amplitude mEPSCs. However, it remains unknown how long this amplitude increase can last and whether it is simply a byproduct of greater release probability. Using *in vitro* patch clamp recording on acute rat brain slices, we found that a brief, high frequency stimulation (HFS) of afferents induced a potentiation of mEPSC amplitude lasting up to 20 min. This amplitude potentiation did not correlate with changes in mEPSC frequency, suggesting that it does not reflect changes in presynaptic release probability. Nonetheless, neither postsynaptic calcium chelator nor the NMDA receptor antagonist blocked the potentiation. Together with the known calcium dependency of HFS-induced potentiation of mEPSCs, our results imply that mEPSC amplitude increase requires presynaptic calcium. Further analysis showed multimodal distribution of mEPSC amplitude, suggesting that large mEPSCs were due to multivesicular glutamate release, even at late post-HFS when the frequency is no longer elevated. In conclusion, high frequency activation of excitatory synapses induces lasting multivesicular release in the SON, which is independent of changes in release probability. This represents a novel form of synaptic plasticity that may contribute to prolonged excitatory tone necessary for generation of burst firing of magnocellular neurons**.**

## Introduction

Magnocellular neurons (MCNs) of the supraoptic nucleus (SON) send their axon terminals to the posterior pituitary where, upon appropriate physiological stimulation, oxytocin (OT) and vasopressin (AVP) are released into the bloodstream. This expulsion of hormone into the periphery is known to be coupled to the electrical activity of MCNs [1]. It is also known that activation of glutamatergic receptors is essential for generating the characteristic burst firing activity of MCNs that optimizes release of AVP and OT [2–5]. Thus, glutamate relays important physiological information when there is a need for AVP and/or OT release, including dehydration, lactation or parturition.

Excitatory synapses on MCNs display a unique plasticity characterized by a lingering barrage of spontaneous transmission that is capable of inducing slow depolarization and prolonged after-discharge of the postsynaptic neuron [6,7]. Accordingly, brief high frequency stimulation (HFS) of afferent fibers to MCNs induces a robust increase in the frequency of tetrodotoxin (TTX)-insensitive miniature EPSC (mEPSC) that lasts for tens of minutes [7,8]. This occurs in the absence of any change in evoked EPSCs [7]. The amplitude of mEPSCs also increases immediately following HFS, due to multivesicular release [7]. Removal of extracellular calcium completely abolishes any effect of HFS on mEPSCs, suggesting that not only the frequency but also the amplitude response is initiated by calcium influx [8]. It remains unknown, however, how long the amplitude change can last and whether it is simply a byproduct of increased release probability.

Therefore, in the present study we characterized the short-term plasticity of mEPSC amplitude and its underlying mechanism in the SON. We provide evidence that strong synaptic activity can induce delayed multivesicular release up to 20 min in the absence of a change in release probability. Such potentiation of multivesicular release represents a unique form of synaptic plasticity that may contribute to the glutamate-mediated induction and maintenance of typical burst firing activity of MCNs.

## Methods

All experiments in this study were carried out in accordance with guidelines established by the Canadian Council on Animal Care and as approved by the Memorial University Institutional Animal Care Committee (13-03-MH).

### Slice Preparation

Male Sprague-Dawley rats (60-80 g) were deeply anesthetized using halothane or isoflurane prior to decapitation. The brain was rapidly removed and 250 µm thick coronal sections containing the SON were generated in ice-cold buffer solution composed of the following (in mM): 87 NaCl, 2.5 KCl, 1.25 NaH_2_PO_4_, 7 MgCl_2_, 0.5 CaCl_2_, 25 NaHCO_3_, 25 glucose, 30 sucrose, 3 pyruvic acid and 1 ascorbic acid, bubbled with 95% O_2_, 5% CO_2_. Slices were incubated at 33-34°C for 45 minutes and then at room temperature until recording in bubbled artificial cerebrospinal fluid (aCSF) composed of the following (in mM): 126 NaCl, 2.5 KCl, 1.2 NaH_2_PO_4_, 1.2 MgCl_2_, 2 CaCl_2_, 25 NaHCO_3_, 10 glucose, and 1 ascorbic acid.

### Electrophysiological Recording

Slices were hemisected, placed in a recording chamber and perfused at 1.5-2.5 ml/min with aCSF at 27-29°C. Infrared differential interference contrast optics (IR-DIC; DM LFSA, Leica Microsystems) were used to visualize cells in the SON. Whole cell patch clamp recordings were performed on MCNs in the SON with MultiClamp 700B amplifier (Molecular Devices, Sunnyvale, CA). Nystatin was used as a perforating agent to obtain access unless stated otherwise, where conventional whole-cell access was attained. For nystatin perforated patch recording, the pipette solution contained the following (in mM): 120 K-gluconate, 5 MgCl_2_, 10 EGTA and 40 HEPES, pH 7.3. Nystatin was dissolved in dimethyl sulfoxide with Pluronic F127 and added to the internal solution to yield a final concentration of 450 µg/ml. Glass electrodes had a tip resistance of 3-7 MΩ when filled with the internal recording solution. Series/access resistance of 10-40 MΩ was normally attained within less that 20 min after formation of a gigaohm seal (1-8 GΩ). For conventional whole cell recording, internal solution consisted of (in mM): 123 K-gluconate, 2 MgCl_2_, 8 KCl, 0.2 EGTA, 10 HEPES, 4 Na_2_-ATP, 0.3 Na-GTP, pH 7.3. EGTA was increased to 10 mM or replaced with 10 mM BAPTA when necessary.

MCNs were initially identified according to their large size, tight juxtaposition to one another, and location immediately lateral to the optic chiasm. Electrophysiologically, all MCNs were identified based on the characteristic delayed onset to action potential generation in response to a depolarizing current injection [9]. Putative OT neurons were identified by the presence of both an inward rectification as well as a sustained outward rectification to a series of hyperpolarizing steps from more depolarized potential (200 msec steps starting at -40 mV down to -130 mV in 10 mV increments), while putative AVP neurons were classified by an absence of rectification, displaying a linear current-voltage relationship to the same protocol [10,11]. Any cells that had ambiguous characteristics were not included in analysis where phenotypes of MCNs were concerned. Experiments were conducted in voltage-clamp mode at a holding potential of -80 mV in the presence of picrotoxin (50 µM) to isolate EPSCs. Input and access resistance were monitored every minute by applying a 20 mV hyperpolarizing pulse for 100 msec. Cells where these parameters changed by 15% or more were not included in the data analysis.

Bipolar tungsten-stimulating electrode was placed immediately dorsal to the SON in order to apply high-frequency stimulation (HFS; 50 or 100 Hz x 1 sec, once or twice in a 10 sec interval). All cells had a graded evoked synaptic response to increasing stimulus intensity. The stimulus intensity giving 50-70% of the maximum evoked EPSC was used to apply HFS.

Spontaneous EPSCs were completely blocked by 6,7-dinitroquinoxaline-2-3-dione (DNQX; 10 µM), indicating that they were non-NMDA receptor mediated. Spontaneous EPSCs recorded in coronal SON preparation are TTX-resistant as have been shown by a number of reports, suggesting they are equivalent to miniature EPSCs (mEPSC) [7,12,13]. In this study, all recordings were done without TTX since the induction of synaptic potentiation is dependent on action potential firing of the presynaptic fibers [7]. All data were collected using pClamp 9 softwares (Molecular Devices, Sunnyvale, CA). Membrane currents were filtered at 1 kHz, acquired at 2-10 kHz sampling rate, and stored for offline analysis.

For AMPA-induced currents, 10 µM AMPA was bath applied for 5 to 10 sec, and resulting inward current was recorded. AMPA application was repeated at least twice in basal condition, and once every 5 minutes after HFS. Cells that displayed consistent AMPA current (< 5% difference) in basal condition and significant increase in mEPSC amplitude after HFS were included in the analysis.

### Data Analysis

Using MiniAnalysis 6.0 software (Synaptosoft, Decatur, GA), mEPSCs clearly standing out from the background noise were selected. For amplitude and rise time analysis, only synaptic events showing a clearly defined baseline, a peak and a smooth rise slope were used. 10-90 percentage points rise time was defined as the time each mEPSC took to rise from 10% to 90% of peak from baseline. For decay times, defined as the time for the mEPSC peak to decay to 1/e, only events that showed a clear baseline, smooth rise to peak and smooth exponential decay to baseline were included.

Following HFS, mEPSC amplitude and frequency changes were considered back to baseline once they fulfilled two criteria: there had to be three consecutive minutes during which the average of measured values over one minute were between 90-110% of baseline value; and during the same period of time, upon performing the Kolmogorov-Smirnov test, no statistical difference in amplitude or interevent interval existed, as compared to control values.

Data are expressed as mean ± SEM. Statistical comparisons were performed using appropriate tests i.e. Kolmogorov-Smirnov test for individual cells, one-way or two-way ANOVA for group comparisons and linear regression analysis for comparisons between two parameters. A value of *p* < 0.05 was considered significant.

### Drugs

Picrotoxin and D-APV were bath-perfused at final concentrations by dissolving aliquots of stock in aCSF. All compounds were purchased from Sigma-Aldrich (St. Louis, MO), except EGTA from EMD Biosciences, Inc. (San Diego, CA), BAPTA from Invitrogen (Burlington, Canada) and Pluronic F127 from BASF (Ludwigshafen, Germany).

## Results

**Figure 1:**
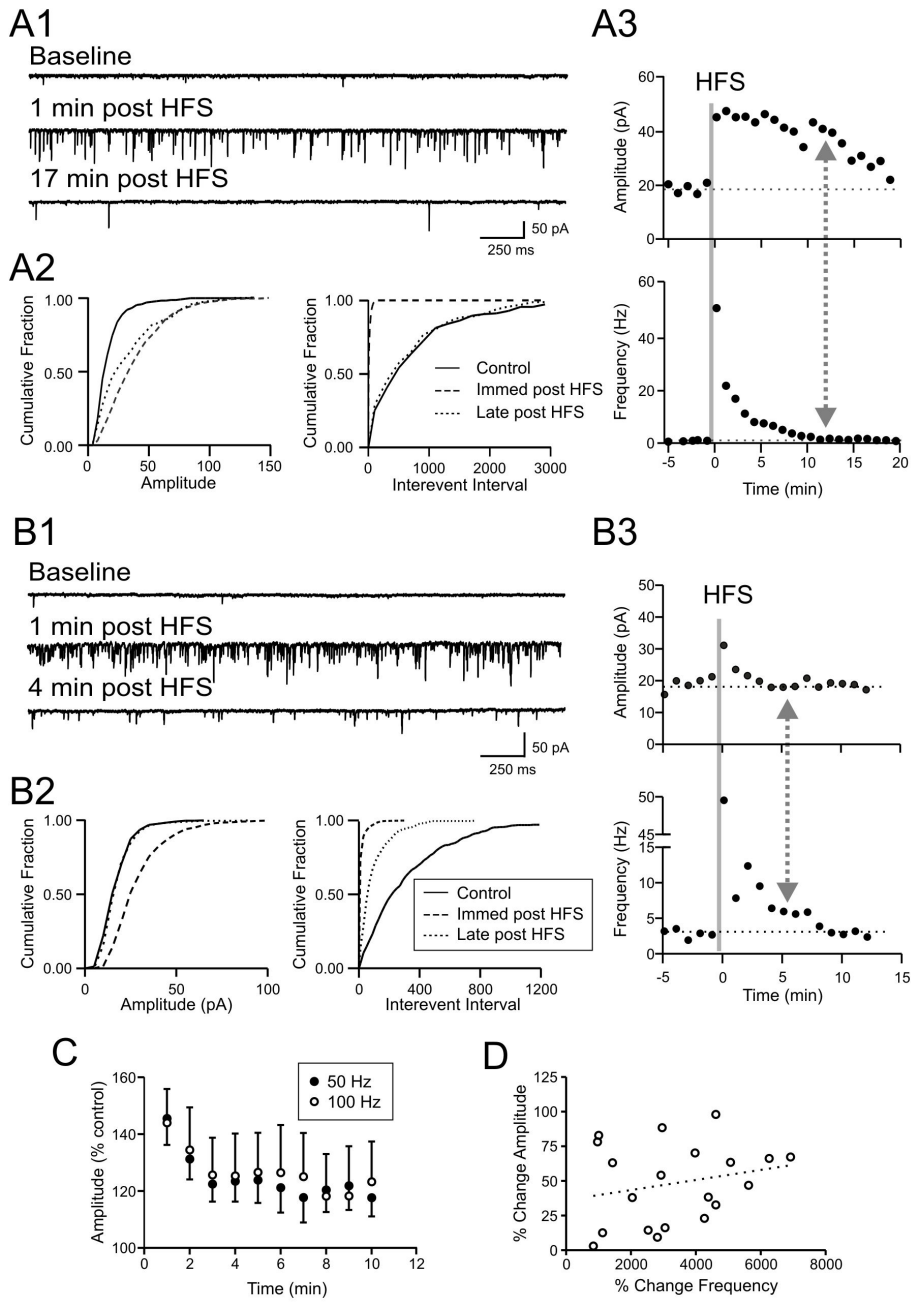
Activity-dependent potentiation of mEPSC amplitude and frequency are dissociated. A1-3) A representative magnocellular neuron that shows an extended mEPSC amplitude potentiation that outlasts the frequency response. A1) Voltage clamp traces taken at time points as indicated. A2) Cumulative plots showing mEPSC amplitude (left panel) and interevent interval (right panel). Late post HFS is after the frequency has recovered. A3) Time-effect plots of mEPSC amplitude (top) and frequency (bottom). Solid vertical line indicates delivery of HFS, dotted vertical line with arrows denotes time when frequency is fully recovered. B1-3) A representative magnocellular neuron that displays an extended potentiation of mEPSC frequency that outlasts the amplitude response. B1) Voltage clamp traces taken at time points as indicated. B2) Cumulative plots of mEPSC amplitude (left panel) and interevent interval (right panel) at time points as indicated. Late post HFS is after the amplitude has recovered. B3) Time-effect plots of mEPSC amplitude (top) and frequency (bottom). Solid vertical line indicates delivery of HFS, dotted vertical line with arrows denotes time when the amplitude is fully recovered. C) Time-effect plot of mEPSC amplitude change in response to 50 Hz, 1 sec (filled circles) or 100 Hz, 1 sec x 2 stimulation (open circles). HFS was applied at time 0. D) Relationship between the initial degree of potentiation for amplitude and frequency (1^st^ min post HFS). Each symbol denotes a cell. There is no significant correlation between the two parameters.

HFS of the afferents at either 50 or 100 Hz induced a significant increase in the amplitude of mEPSCs that typically lasted for several minutes, accompanied by a robust increase in mEPSC frequency that has been characterized previously [7,8]. The time course of the frequency and amplitude changes varied among cells tested. Following HFS, some cells showed an increase in mEPSC amplitude that clearly outlasted the change in the frequency, while in others, the amplitude change returned to baseline levels despite sustained elevated frequency. Figures 1 A1-3 show a representative MCN with a prolonged mEPSC amplitude response as compared to that of the frequency. Figures 1 B1-3 show another MCN that represents a different type of response: a long frequency response that outlasted the amplitude increase. Since HFS at 50 and 100 Hz induced a similar degree of amplitude increase (p>0.05, Figure 1C; n=10 and 11, respectively), data obtained using these protocols were pooled unless stated otherwise. For group comparisons, control and experimental groups used the same stimulation protocol. In control condition, the average basal mEPSC amplitude was 20.3 ± 5.3 pA which was elevated to 30.4 ± 7.1 pA (n = 21) during the first minute post-HFS. Overall, 50% (13 of 26) of MCNs in which mEPSC frequency and amplitude responses were compared showed a longer amplitude response, while the remaining 50% (13 of 26) showed a longer frequency response. No correlation was found between the peak increase of mEPSC amplitude and frequency (% change during the first minute post-HFS) (Figure 1D). Taken together, these results suggest that the potentiation of mEPSC amplitude and frequency are independent of each other.

**Figure 2:**
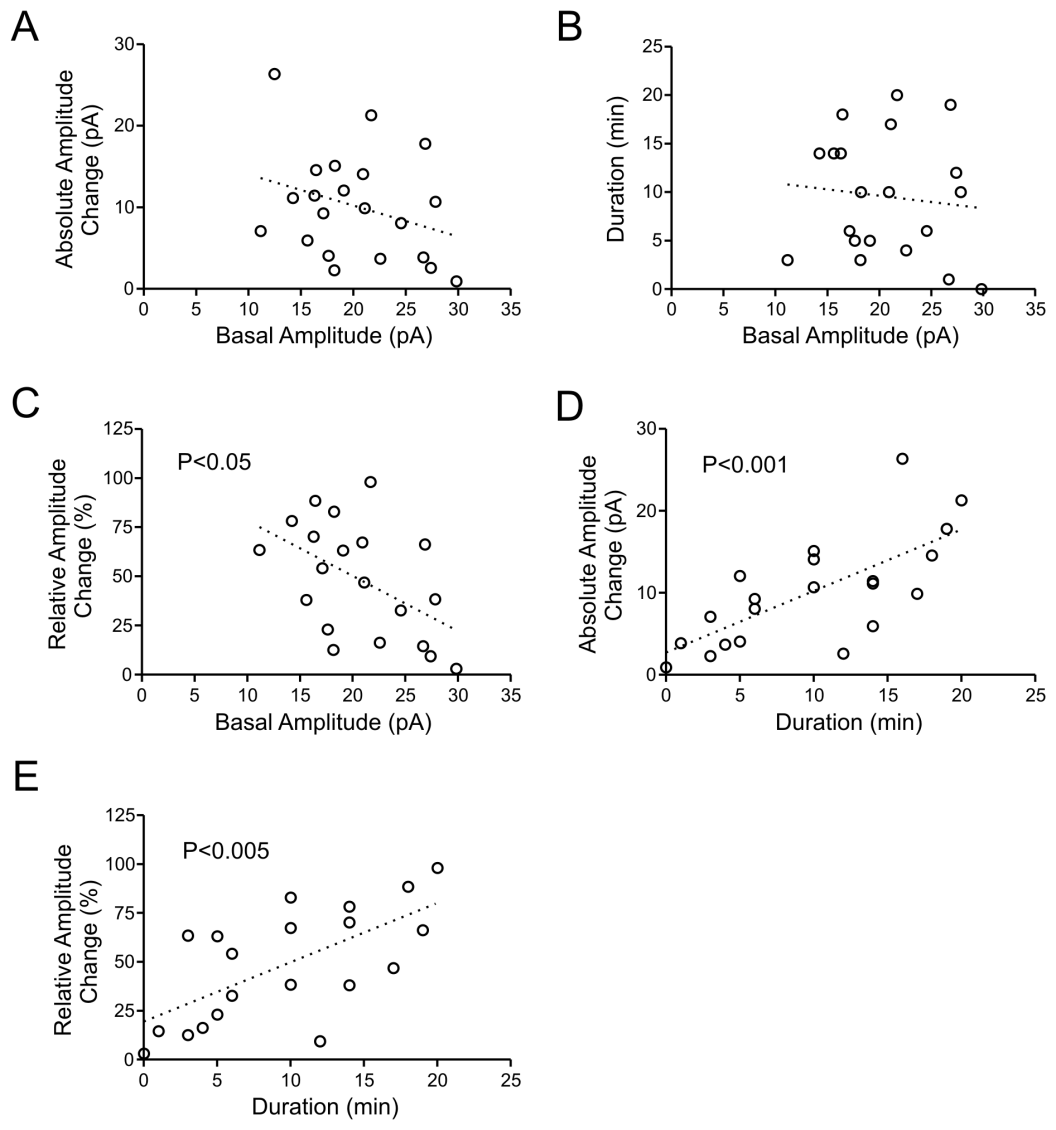
Relationships between basal mEPSC amplitude, peak change and duration of response to HFS. In all graphs in this figure and Figure 3, each circle denotes a single neuron. p values are indicated within graphs if they reached a significance. A-C) Linear regression analysis showing that the basal mEPSC amplitude is not related to the absolute amplitude change during the 1^st^ min post-HFS (A) or duration of the change (B). In contrast, a negative correlation exists between the amplitude in baseline control and 1^st^ min post-HFS conditions upon normalizing the amplitude response to control values (C). D and E) Linear regression analysis showing that both absolute (D) and normalized (relative; E) responses during the 1^st^ min post-HFS are positively related to how long the response persists.

**Figure 3:**
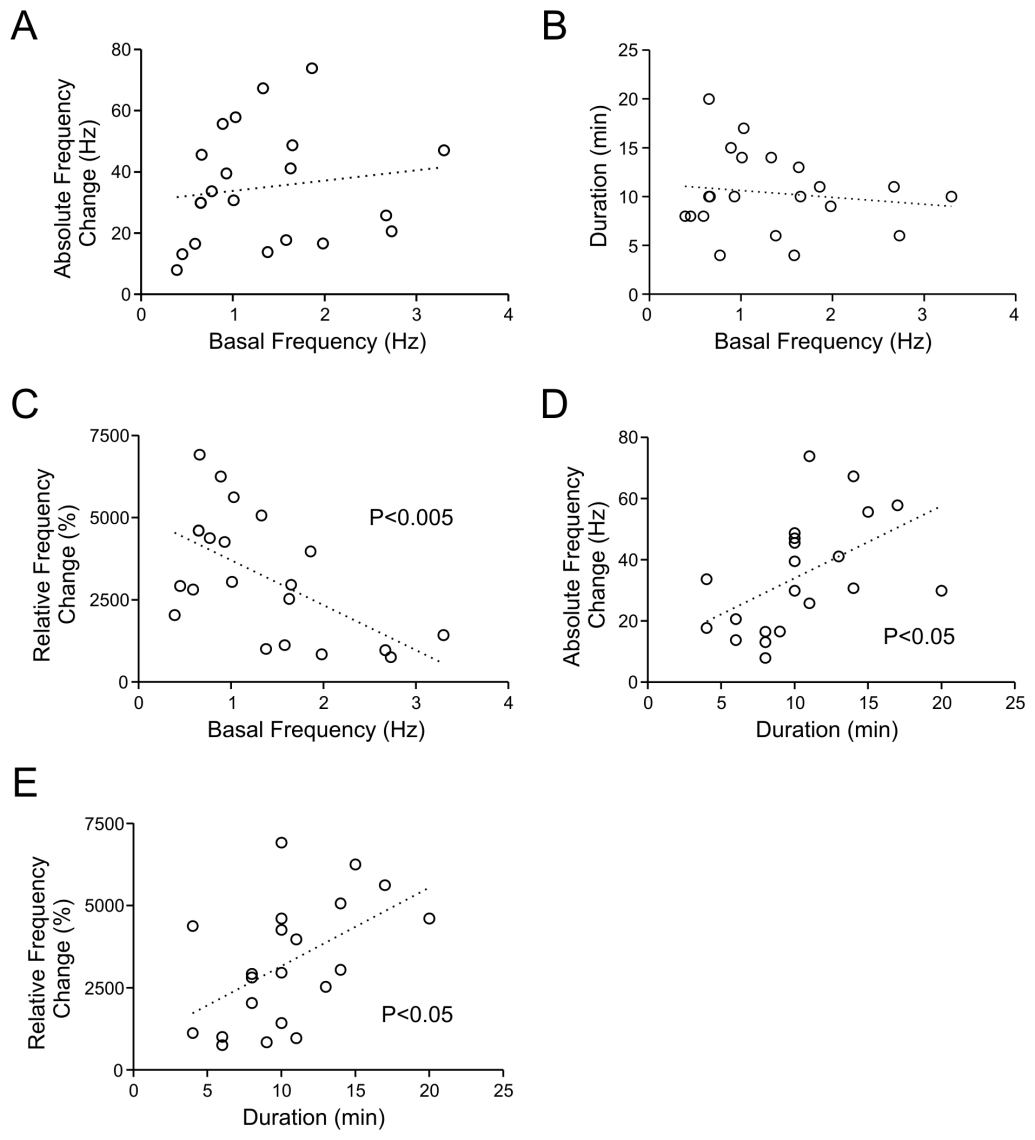
Relationships between basal mEPSC frequency, peak change and duration of response to HFS. A-C) Linear regression analysis showing that the basal mEPSC frequency is not related to the absolute frequency change during the 1^st^ min post-HFS (A) or duration of the change (B). In contrast, there is a negative correlation between the control baseline values and the changes normalized to control (C). C and D and E) Linear regression analysis showing that both absolute (D) and normalized responses (E) during the 1^st^ min are positively related to how long the response persists. E) Linear regression analysis showing no relationship between the basal mEPSC frequency and the duration of response.

Since half of the cells demonstrated an extended frequency response while the other half demonstrated an extended amplitude response, we asked whether this could be due to the neurochemical phenotype of MCNs (AVP vs. OT) or the stimulation protocol. This seems not to be the case, as neither the neurochemical phenotype nor the HFS frequency (50 vs. 100 Hz) was associated with specific form of synaptic plasticity, as summarized in Table 1.

**Table 1:** Number of magnocellular neurons examined that displayed extended increase in mEPSC amplitude (AMP) or frequency (FREQ) following high frequency stimulation.

		Extended AMP	Extended FREQ	Total
Neurochemical phenotype	AVP	5 (50.0%)	5 (50.0%)	10
	OT	4 (44.4%)	5 (55.6%)	9
Stimulation protocol	50 Hz	8 (50.0%)	8 (50.0%)	16
	100 Hz	5 (55.6%)	4 (44.4%)	9

% in brackets indicates the proportion of cells in each category.

Next, we asked whether the basal condition affected the degree of potentiation (both peak increase and duration). It might be possible that when the synaptic events are small in basal condition, there is more capacity for potentiation. However, we found no correlation between the basal amplitude and its absolute peak increase during the first minute following HFS (Figure 2A) or the total duration of STP (Figure 2B), suggesting that the initial amplitude did not affect how much or how long it would potentiate (i.e. no ceiling effect). In contrast, normalizing the amplitude increase to baseline values revealed a negative correlation with the basal amplitude (Figure 2C), indicating that weaker synaptic inputs in control condition result in a proportionally larger degree of potentiation. We also found a positive correlation between the duration and the peak potentiation (both absolute and relative amplitude change) (Figure 2D and E). Comparable analyses were performed on mEPSC frequency before and after HFS, and we found similar pattern of correlations between basal frequency, initial magnitude and duration of frequency potentiation (Figure 3A-E).

**Figure 4:**
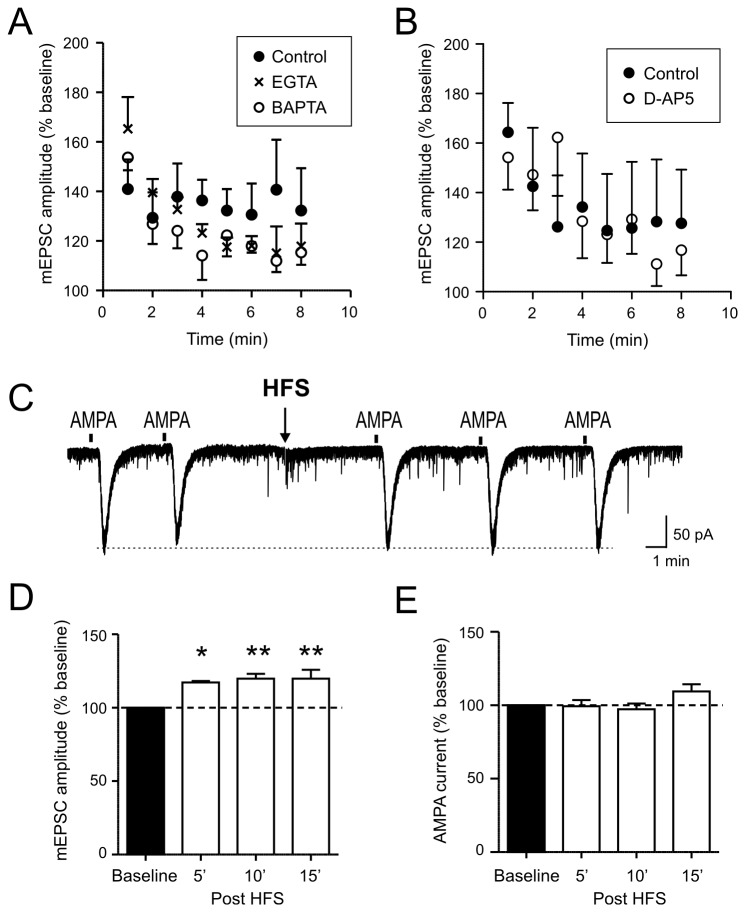
HFS-induced mEPSC amplitude increase is not dependent on postsynaptic calcium or NMDA receptors. A) Time effect plot of amplitude showing that 10 mM BAPTA or EGTA included in the recording pipette fails to block the amplitude response (open circles, BAPTA; crosses, EGTA; filled circles, whole cell control). B) Time effect plot of amplitude showing that 25 µM D-APV fails to block the amplitude response (open circles, D-APV; filled circles, control). C) A representative voltage clamp trace showing inward currents induced by brief bath applications (10 sec) of 10 µM AMPA. The stimulus artifact during HFS has been blanked for clarity of presentation. D) In magnocellular neurons tested for AMPA current responses, average mEPSC amplitude is significantly greater post-HFS up to 15 min, compared to that during baseline. *p<0.05, **p<0.01. E) In contrast, AMPA current amplitude does not change before and after HFS. D and E show data from the same experiments.

For the remainder of the study, we focused on the potentiation of mEPSC amplitude, which had not been well characterized. First, we sought to determine whether the prolonged increase in mEPSC amplitude was due to a pre- or postsynaptic mechanism. We previously showed that removing external calcium completely abolished any amplitude change induced by HFS [8]. In order to examine the role of calcium in the postsynaptic cell, 10 mM BAPTA (n=6) or EGTA (n=3), fast-acting or high-capacity calcium chelator, respectively, was included in the intracellular pipette solution and recordings were done with conventional whole cell mode. Neither chelator blocked the effect of HFS on mEPSC amplitude: its magnitude and duration were comparable to the whole-cell control group (n=6) (p>0.05, Figure 4A). We also tested the effect of the NMDA receptor antagonist D-APV, which has been shown to block postsynaptic long-term potentiation of evoked EPSCs in the SON [14]. In MCNs treated with D-APV (25 µM) for 20 minutes (n=5), HFS was followed by a potentiated mEPSC amplitude to a similar extent as control (n=5) (p>0.05, Figure 4B). Furthermore, to test for a change in AMPA receptor sensitivity, response to brief exogenous application of AMPA (10 µM, 5 or 10 sec) was examined. Despite the significant increase in mEPSC amplitude post HFS, there was no change in the amplitude of AMPA-induced current (n=4, Figure 4C-E). These results provide definitive evidence that the locus of the amplitude potentiation is at the presynaptic terminal.

**Figure 5:**
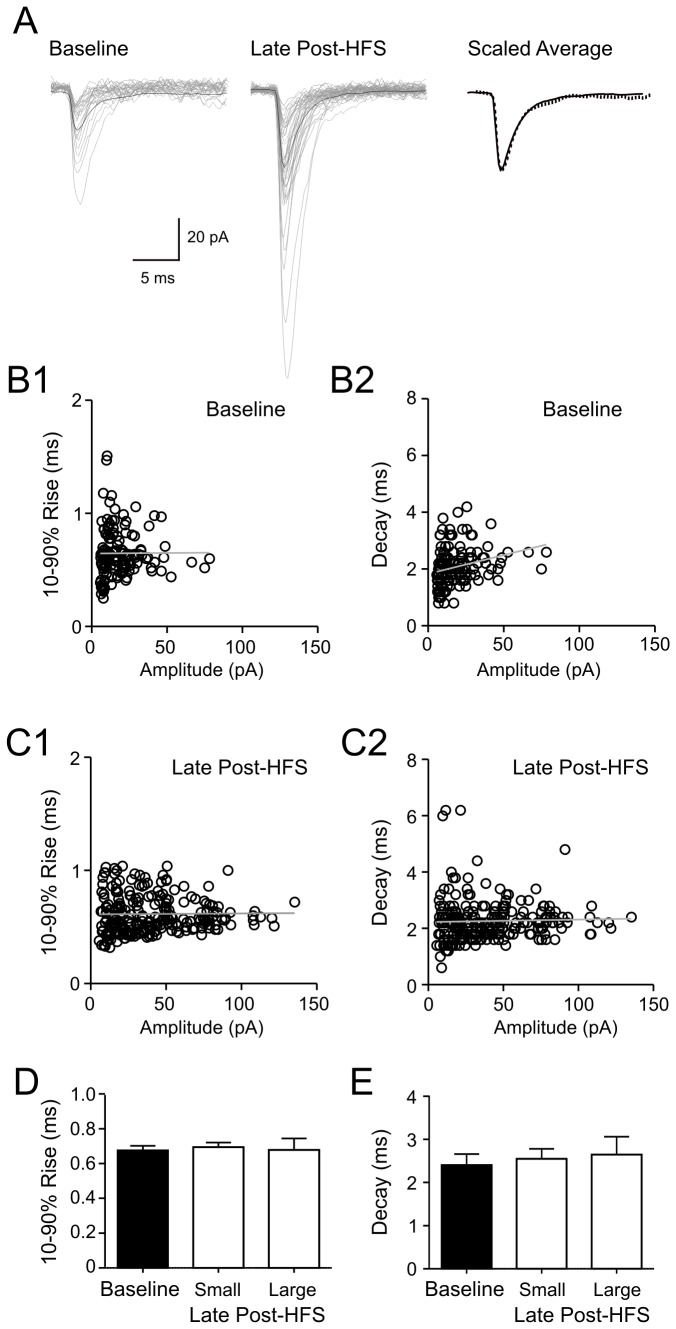
mEPSCs before and after HFS have similar kinetics. A) Superimposed mEPSCs (grey lines) from control (left panel) and late post-HFS (middle panel) with averaged traces shown in black. Right panel: averaged traces, scaled and superimposed (solid line: control; dotted line: late post-HFS). B) Characteristics of mEPSCs recorded from a representative cell during baseline condition. 10-90 percentage points rise (B1) or decay times (B2) are plotted against their amplitudes. Each circle indicates individual mEPSC. Linear regression analysis indicates no relationship between the factors (p>0.05). C) Scatter plot of 10-90% rise (C1) or decay times (C2) vs. amplitudes of mESPCs recorded from the same cell as (B) at late post-HFS after frequency has recovered to baseline. Linear regression analysis indicates no relationship between the factors (p>0.05). D and E) Summary graphs showing 10-90% rise (D) and decay (E) of mEPSCs in baseline and late post-HFS. Post-HFS mEPSCs are grouped according to the amplitude as small (< 20 pA) or large (> 45 pA). D and E show data from the same group of cells.

A dissociation of mEPSC amplitude and frequency responses provided us with an opportunity to investigate the potentiation of mEPSC amplitude in isolation from the plasticity of mEPSC frequency. mEPSCs were analyzed in cells that had a longer amplitude potentiation after the frequency had returned to baseline (late post-HFS). We first asked whether HFS activates previously quiescent synapses that would give rise to postsynaptic responses with large amplitude and distinct kinetics. However, we found that mEPSCs that appeared in control and late post-HFS showed similar rise and decay, which had no correlation with the amplitude of mEPSCs (Figure 5A-C). In six cells examined, there was no statistical differences in the rise or decay time of mEPSCs recorded during baseline and late post-HFS, regardless of the amplitude (p>0.05, Figure 5D and E).

**Figure 6:**
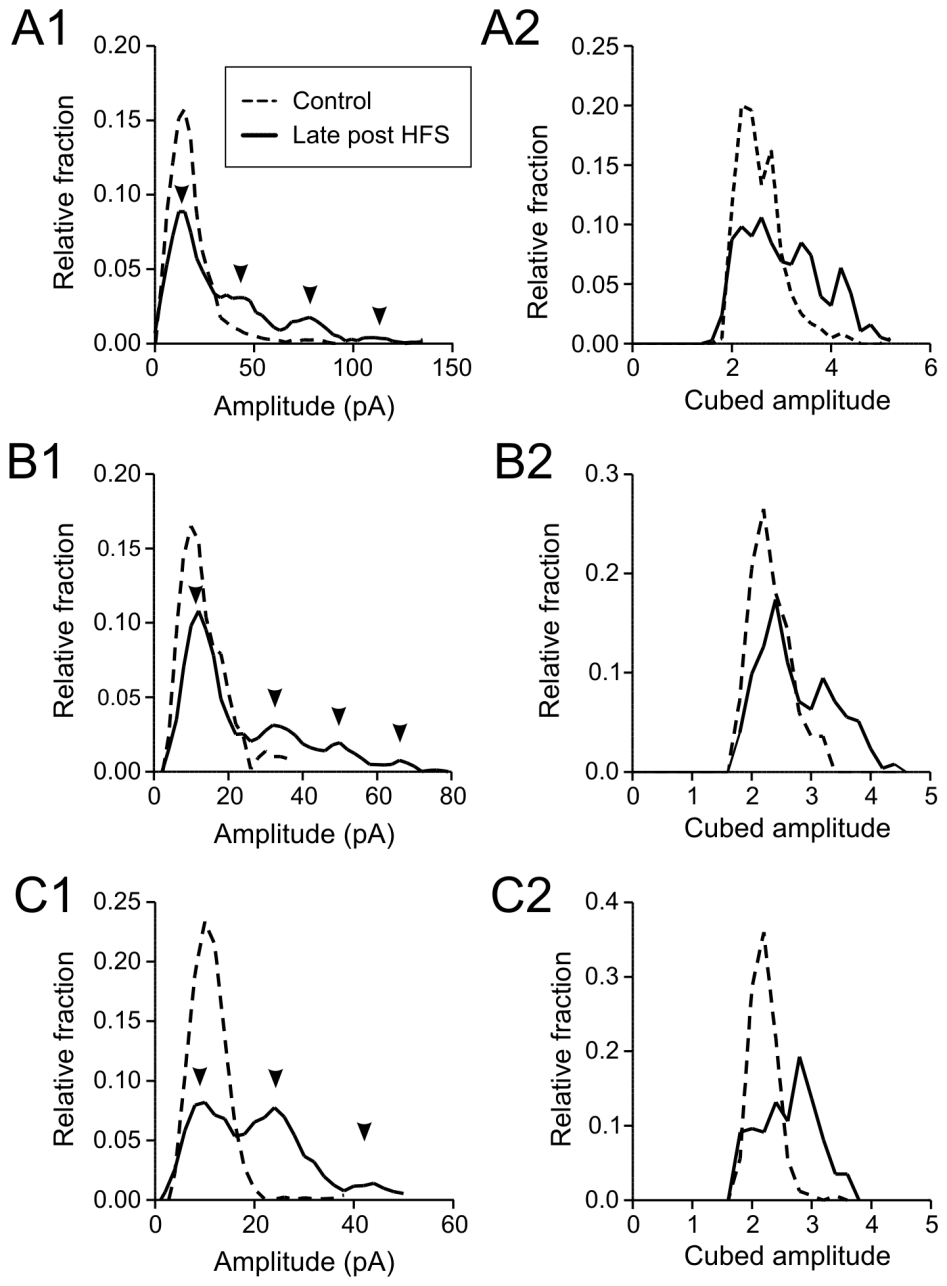
Multivesicular transmitter release underlies large mEPSCs. A1, B1 and C1) Amplitude distribution histograms from three magnocellular neurons. Data from baseline control (dotted line) and late post-HFS after frequency response has ended (solid line) are superimposed. Note that peaks in late post-HFS (arrowheads) are approximately equidistant and the peak in control distribution is approximately the same size as the first peak in late post-HFS. A2, B2 and C2) Cubed root transformation of mEPSC amplitude shown in A1, B1 and C1, respectively. Multiple peaks are still apparent.

Additional examination of the amplitude distribution histograms revealed clear multiple peaks that are approximately equidistant from each other in the late post-HFS period in 6 out of 10 cells examined (Figure 6A1, B1 and C1). This suggests that large mEPSCs are due to multiquantal release. In contrast, variation of quantal size is unlikely to be a major factor for the skewness in the amplitude distribution, as cubic root transformation of mEPSC amplitude did not reveal a Gaussian distribution (Figure 6A2, B2, and C2).

## Discussion

The present study demonstrates that an intense but brief activation of excitatory synapses to the SON results in the potentiation of mEPSC amplitude lasting up to 20 minutes, due to multivesicular release of glutamate from excitatory terminals. Evidence that points to the presynaptic terminals as the site of synaptic potentiation comes from our previous study showing the dependence of synaptic potentiation on extracellular calcium [8], together with the present study demonstrating a lack of effect of postsynaptic BAPTA and EGTA. These data suggest that calcium influx into the presynaptic terminals, but not the postsynaptic cell, is a requirement. To further support this idea, the amplitude change is insensitive to the NMDA receptor antagonist. This is in contrast to HFS-induced long-term potentiation of evoked EPSCs in the SON which occurs postsynaptically and is blocked by NMDA receptor antagonists or postsynaptic calcium chelator [14]. Therefore, a short-term potentiation of mEPSC amplitude represents another form of synaptic plasticity that these SON synapses are capable of experiencing. A lack of NMDA receptor involvement also eliminates the possibility of HFS inducing NMDA receptor-dependent release of retrograde messengers from neighboring cells, which in turn could act on the excitatory synapses to affect transmitter release. In addition, an increase in mEPSC amplitude does not accompany any change in AMPA-induced current, further supporting the presynaptic mechanism.

We found that the time course and magnitude of mEPSC amplitude potentiation do not correlate with those of the frequency change [7,8]. Thus, the potentiation of mEPSC amplitude occurs independently of that of mEPSC frequency. We took advantage of the cells that displayed a longer change in the amplitude than frequency to study the plasticity of mEPSC amplitude in isolation. By comparing mEPSC amplitude using cubed root transformation in baseline condition and those that appeared late post-HFS after frequency had returned to baseline, we determined that variation in vesicle size is not a major factor underlying skewed amplitude distributions. This contrasts to a report on MCNs in the paraventricular nucleus, where mEPSC amplitude variation has been attributed only to vesicle size [15].

We also found that HFS induced no significant change in the kinetics of mEPSCs (10-90% rise and decay) despite the differences in the amplitude. This excludes the possibilities of previously quiescent proximal synapses becoming active and giving rise to large mEPSCs [16] or spillover of glutamate summating onto ongoing activity in a neighboring synapse [17], as these should alter the kinetics of postsynaptic responses. The fact that larger EPSCs appear while the frequency has returned to its original value further supports this, since activation of additional synapses would be expected to increase the frequency. It is reasonable to think that the synapses contributing to mEPSCs in baseline conditions are also responsible for large mEPSCs post-HFS. A random summation of quantal events can also result in large synaptic currents; however, in order to constitute large mEPSCs with smooth rise to peak, transmission at multiple synapses would have to completely coincide. The probability of this happening so frequently and consistently while mEPSCs are occurring at overall frequency of 1-3 Hz is extremely low. Altogether, these led us to conclude that HFS-induced large mEPSC is due to coordinated multivesicular release at single release site. This is supported by the amplitude distribution histogram of late post-HFS events displaying equidistant multimodal peaks. Based on the number of peaks, large mEPSCs are equivalent to two to four quanta at the SON synapse.

Our results imply that multivesicular release at the SON excitatory synapse is not due to high release probability, as there is no correlation between the potentiation of mEPSC frequency and amplitude. This is in contrast to other synapses where high release probability promotes multivesicular release, such as ribbon synapses in the retina and Schaffer collateral – CA1 synapses [18,19]. In contrast, multivesicular release is not influenced by release probability at other synapses such as inner hair cell synapse [20,21]. Therefore, different synapses have different mechanisms for controlling multivesicular release.

Which change outlasts the other, the amplitude or frequency, appears to be random and independent of the postsynaptic phenotype or stimulation intensity. Given the presynaptic origin of these changes, the property of presynaptic terminals involved may be the decisive factor. It is possible that different presynaptic terminals show distinct forms of synaptic plasticity in response to HFS, and what we observe is a sum of these effects because our stimulating electrode would activate many presynaptic fibers. Indeed, the source of glutamatergic projections to the SON is diverse, including intra- and extrahypothalamic brain regions [13,22–24]. The organum vasculosum lamina terminalis (OVLT) is one of the prominent sources of glutamatergic afferents to the SON to signal osmotic information. Hyperosmotic stimulation of the OVLT results in a barrage of asynchronous glutamate release onto SON MCNs [25]. A 10 Hz-train stimulation of the OVLT induces a prolonged depolarization and after-discharge of MCNs in the SON that outlast the train and are blocked by glutamate receptor antagonists [22]. These characteristics of OVLT-SON synapses are similar to those of the HFS-induced short-term synaptic plasticity of mEPSCs described here.

MCNs are known for their simple dendritic trees and high input resistance [26,27]. Thus, mEPSCs are capable of causing significant depolarization and generating action potentials under appropriate conditions [7,28,29]. Potentiation of mEPSP amplitude would increase the chance of the postsynaptic cell reaching the threshold. This would increase the frequency and fluctuation of basal firing activity which in turn could promote burst firing [30,31]. An increase in mEPSP frequency can also be expected to elevate the overall level of excitation [32]. A comparative analysis of basal condition and HFS-induced mEPSCs revealed that the baseline frequency and amplitude do not influence the extent of response to HFS. This suggests that in our preparation, the excitatory synapses in the SON are nowhere near their capacity limit and are amenable to potentiation upon appropriate stimuli. Also this means that cells having smaller or less frequent mEPSCs will experience a relatively larger impact of the change. This may help relatively inactive cells become active in synchrony with other cells.

On the whole, activity-dependent plasticity of mEPSCs in the SON signifies a memory trace of recent synaptic activity. It is a cost-efficient mechanism without the involvement of somatic activity. This provides a modest but continuous excitatory drive to the postsynaptic MCNs, in which a prolonged depolarization triggers neuropeptide release more efficiently than precisely timed and isolated synaptic input.
